# Unravelling the role of host plant expansion in the diversification of a Neotropical butterfly genus

**DOI:** 10.1186/s12862-016-0701-5

**Published:** 2016-06-16

**Authors:** Melanie McClure, Marianne Elias

**Affiliations:** Institut de Systématique, Évolution, Biodiversité, ISYEB - UMR 7205 – CNRS, MNHN, UPMC, EPHE, Muséum National d’Histoire Naturelle, Sorbonne Universités, 57 rue Cuvier, CP50, F-75005 Paris, France

**Keywords:** Adaptive divergence, Aposematic mimicry, Diet breadth, Ecological speciation, Host shift, Niche width, Oviposition preference, Radiation, Resource use

## Abstract

**Background:**

Understanding the processes underlying diversification is a central question in evolutionary biology. For butterflies, access to new host plants provides opportunities for adaptive speciation. On the one hand, locally abundant host species can generate ecologically significant selection pressure. But a diversity of host plant species within the geographic range of each population and/or species might also eliminate any advantage conferred by specialization. This paper focuses on four *Melinaea* species, which are oligophagous on the family Solanaceae: *M. menophilus*, *M. satevis*, *M. marsaeus*, and finally, *M. mothone*. We examined both female preference and larval performance on two host plant species that commonly occur in this butterfly’s native range, *Juanulloa parasitica* and *Trianaea speciosa*, to determine whether the different *Melinaea* species show evidence of local adaptation.

**Result:**

In choice experiments, *M. mothone* females used both host plants for oviposition, whereas all other species used *J. parasitica* almost exclusively. In no choice experiment, *M. mothone* was the only species that readily accepted *T. speciosa* as a larval host plant. Larval survival was highest on *J. parasitica* (82.0 % *vs.* 60.9 %) and development took longer on *T. speciosa* (14.12 days *vs.* 13.35 days), except for *M. mothone*, which did equally well on both host plants. For all species, average pupal weight was highest on *J. parasitica* (450.66 mg *vs.* 420.01 mg), although this difference was least apparent in *M. mothone*.

**Conclusion:**

We did not find that coexisting species of *Melinaea* partition host plant resources as expected if speciation is primarily driven by host plant divergence. Although *M. mothone* shows evidence of local adaptation to a novel host plant, *T. speciosa*, which co-occurs, it does not preferentially lay more eggs on or perform better on this host plant than on host plants used by other *Melinaea* species and not present in its distributional range. It is likely that diversification in this genus is driven by co-occurring Müllerian mimics and the resulting predation pressure, although this is also likely made possible by greater niche diversity as a consequence of plasticity for potential hosts.

## Background

Understanding the processes underlying diversification is a central question in evolutionary biology. In butterflies, access to new host plants provides opportunities for adaptive speciation, and is thought to be the primary driving mechanism for the diversification of phytophagous insects [1]. Indeed, there exists a positive correlation between host plant diversity and butterfly species diversity in general [2]. Janz et al. [2] postulated that the diversification of phytophagous insects has been driven by oscillations in host plant ranges, which can lead to increased species distributions and subsequently opportunities for secondary specialization on novel hosts (see also [3]). Although multiple-host use implies increased plasticity, which should counterbalance local adaptation, Janz & Nylin [4] suggested that host expansions can also lead to other factors causing divergent selection to increase population fragmentation and for diversification to occur.

As such, locally abundant host plant species might generate ecologically significant selection pressures, and adaptive evolution in response to locally abundant plant species has been shown to occur, even in the face of high gene flow. For example, Eastern tiger swallowtail butterflies (*Papilio glaucus*) showed greater oviposition preference and larval performance on host plants that were more abundant at their collection sites [5]. However, a diversity of host plant species within the geographic range of each population and/or species can also eliminate any advantages conferred by specialization. For example, Ladner & Altizer [6] found that although host plant influenced oviposition and larval performance in the Monarch (*Danaus plexippus*), they could find no evidence for local adaptation to hosts found within each of the butterfly population’s breeding range. Indeed, Chew [7] noted that species that lay eggs singly and visit many different host species might be slow to evolve consistent preferences.

The genus *Melinaea* belongs to the Neotropical tribe Ithomiini (Nymphalidae: Danainae), which are aposematic butterflies extensively involved in Müllerian mimicry rings. Butterfly species that are part of a Müllerian mimicry ring possess the same warning signal, and effectively share the cost of educating predators of their unpalatability. In the genus *Melinaea*, species often consist of multiple subspecies characterised by different wing colour patterns which are associated with distinct mimetic communities. The systematics for this genus remains unclear. Previous studies using mitochondrial and nuclear genes, and rapidly evolving microsatellite markers, show little genetic differentiation among taxa, which suggests recent speciation with incomplete lineage sorting, possibly combined with recent gene flow [8–11]. One possible explanation for this rapid diversification is radiation across different host plants. Records of host plant usage suggest that *Melinaea* are oligophagous on the family Solanaceae, specifically the widespread tribe Juanulloeae, which might be the reason for their large geographic distribution [12]. However, different populations of different species are likely to encounter only a subset of potential hosts, as no single host plant overlaps the entire geographic range. For example, in Ecuador, *M. menophilus* has been shown to use both *Juanulloa ochracea* (K. Willmott, pers. com.) and *Juanulloa mexicana* [13] in the lowlands, but *Markea* sp. [14] in the montane. Because many environmental variables are correlated with elevation, adaptation and divergent selection have been shown to occur along altitudinal ranges (e.g., [15]).

Studying host plant choice in the genus *Melinaea* may provide insights into how adaptation can influence the diversification of mimetic butterflies. For example, detailed studies of heliconiine communities demonstrate that coexisting species of *Heliconius* partition host plant resources [16], as expected if speciation is driven by host plant divergence. However, speciation in *Heliconius* has also been shown to be possible without host shifts [17], and competitive exclusion on larval hosts may instead be driving the observed patterns of distribution. The *Melinaea* species in north-eastern Peru (San Martín and Loreto department) are of particular interest for such studies, as multiple species, many consisting of different subspecies, are present and overlap in distribution (see [11]).

Here we examine whether the different *Melinaea* species show evidence of local adaptation to host plant species that commonly occur in their native range. Some herbivores are known to feed on plants for which larval performance (i.e., survivorship and development time) is suboptimal, raising the possibility that other extrinsic factors, such as competition or enemy-free space, may contribute to insect fitness and influence the evolution of diet breadth [18]. As such, we look at both female preference and larval performance. We predict that exposure to different host plant species in their respective distribution ranges could select for divergent host use traits, so that butterflies should preferentially lay more eggs on, and larvae should perform better on, species common to their native habitats. We also attempt to determine whether the directionality of larval performance corresponded with female oviposition preference.

## Methods

This paper focuses on four *Melinaea* species: *M. menophilus* (which consists of the two subspecies *M. menophilus* ssp. n 1 and *M. m. hicetas*, found in transitional forest and lowland forest respectively), *M. mothone*, which is found in high altitudinal habitats, *M. marsaeus* (consisting of the two subspecies *M. marsaeus phasiana* and *M. m. rileyi*, found in transitional forest and lowland forest respectively), and *M. satevis cydon*, a lowland species. In a previous study [11], we extensively surveyed host plant use in different habitats and found that all the different *Melinaea* species in this study utilize *Juanulloa parasitica* (Solanaceae) as a host plant, except for *M. mothone*, which uses the novel host plant *Trianaea speciosa* (Solanaceae). Indeed, because *J. parasitica* did not co-occur with *M. mothone*, this butterfly species was never collected on this host plant. Similarly, because *T. speciosa* is only present at higher altitudes, it is present throughout *M. mothone*’s distribution, but only co-occurs with *M. menophilus* ssp. n 1 at the very edge of its distribution, and does not co-occur with any of the other *Melinaea* species in this study. Furthermore, the only species ever collected from *T. speciosa* is *M. mothone*, even when *M. menophilus* was present at low densities. As such, there appears to be an ecological difference related to altitude and host plant use, in addition to genetic differences [11], between *M. mothone* and the other *Melinaea* species of this study. Because the different subspecies of both *M. menophilus* and *M. marsaeus* share similar habitat preference, host plant use, and have overlapping distribution [11], comparisons were only made between the species rather than between the subspecies (i.e., subspecies were pooled).

Gravid wild caught female butterflies were collected in north-eastern Peru (San Martín and Loreto department), in 2012–2013. Collection localities consisted of transitional forest habitats surrounding Tarapoto (Rio Shilcayo basin: 6°27’30”S 76°21’00”W alt 460 m), Carachamera (6°25’85”S 76°15’27”W alt 280 m) and Shapaja (6°36’56”S 76°09’61”W alt 195 m); high altitudinal habitat in the Cordillera Escalera near Tarapoto (the Tunel ridge: 6°27’11”S 76°17’11”W alt 1090 m) and in Moyobamba (6°04’34”S 76°57’27”W alt 1130 m); and lowland forest on Pongo-Baranquita road (6°17’53”S 76°14’38”W alt 200 m) and Shucushyacu (5°57’20”S 75°53’06”W alt 183 m). Because each species has a limited distribution, butterflies of a given species were only collected at a limited number of sites, often in close proximity to each other. Furthermore, previous studies have failed to find any geographical population structure [11]. As such, butterflies from different collection sites were pooled for the analysis. Females were kept in 2 × 2 × 2 m outdoor insectaries under ambient conditions in Tarapoto, San Martín, and were provided with sugar water solution and bee pollen for nourishment.

To test for female oviposition preference across different hosts, we performed two types of trials, as follows: 1) choice experiments, in which a single mated female butterfly was placed in a cage containing both a potted *J. parasitica* and a *T. speciosa*, and 2) no-choice experiments, in which females were placed in cages with two *T. speciosa* plants. For all trials, care was taken so that the plants were of similar size, and that the plants did not touch. No-choice oviposition experiments examined whether female butterflies can accept the novel host plant *T. speciosa* when no alternatives are present. Strict preference of normal hosts in a choice situation does not preclude the possibility of accepting a novel host when no alternative is present. All trials lasted for four days and 18 females per species were tested for each treatment.

The number of eggs laid on each of the plants was recorded at the same time daily and larvae were collected shortly after hatching. Larvae collected from at least eight different unrelated females used in the preference experiment (so as to reduce any maternal or family effects) per species were used for tests of larval performance (*N* = 32/species/host plant). Larvae were reared individually in transparent plastic containers in the shade behind a nearby building under ambient conditions. Leaves of either *J. parasitica* or of *T. speciosa* were offered *ad libitum*, and larvae were checked daily for food replacement and cleaning, as well as survival. Duration of larval development was recorded and pupae were weighed the morning following pupation using a small portable scale.

A generalized linear model (GzLM) was done using SPSS (SPSS Inc., Chicago, Illinois) to determine if the number of eggs laid on either *J. parasitica* or *T. speciosa* in the choice experiment was affected by either the *Melinaea* species tested or the host plant, and to test for a possible interaction between these two factors. Because the number of eggs for most of the species included many zero counts, a negative binomial distribution was used. The total number of eggs laid on both *T. speciosa* in the no-choice experiment was compared between *Melinaea* species using a non-parametric Kruskal-Wallis test. Larval survival was compared between species for each host plant using a multivariate Cox proportional hazards model. Duration of larval development and pupal weight were compared between species and host plants using a 2-way ANOVA to test for an overall effect of treatment.

## Results

**Fig. 1:**
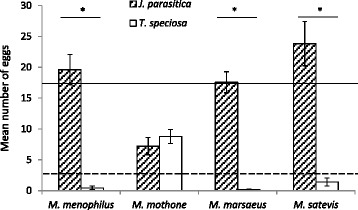
The number of eggs laid (Mean ± SE) on either *Juanulloa parasitica* or *Trianaea speciosa* (choice experiment) by four different *Melinaea* species (*M. menophilus*, *M. mothone*, *M. marsaeus*, and *M. satevis*; *N* = 18 females/species). *Full and dash lines* indicate average number of eggs laid on *J. parasitica* and *T. speciosa* respectively, for all species combined. * indicates significant difference (*p* < 0.001) between host plants for each species

In the choice experiment, the number of eggs laid was not significantly different for the different *Melinaea* species (Wald’s *χ*^2^ = 2.334; df = 3; *p* = 0.506), but it was significantly affected by host plant (Wald’s *χ*^2^ = 44.816; df = 1; *p* < 0.001), and there was a significant interaction between the two factors (Wald’s *χ*^2^ = 140.997; df = 5; *p* < 0.001; Fig. 1). This was due to *M. mothone*, which laid 60 % of their eggs on average on *T. speciosa*, whereas the average for all *Melinaea* species combined was of 82.3 % of eggs on *J. parasitica vs.* 17.7 % on *T. speciosa.* The other species showed virtually identical patterns of oviposition behaviour, laying almost all of their eggs on *J. parasitica* (Fig. 1).

**Fig. 2:**
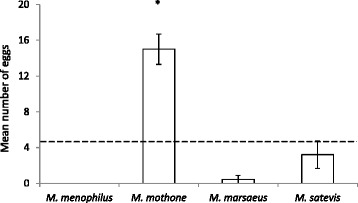
The total number of eggs (Mean ± SE) laid on both of the *Trianaea speciosa* plants (no choice experiment) by four different *Melinaea* species (*M. menophilus*, *M. mothone*, *M. marsaeus*, and *M. satevis*; *N* = 18 females/species). *Dash line* indicates average number of eggs laid on *T. speciosa* across species. * indicates *Melinaea* species that are significantly different from the others (*p* < 0.001)

Similarly, in the no choice experiment, the mean rank for the number of eggs *M. mothone* laid on the two *T. speciosa* plants (58.85) was much higher than that of the other species (*M. menophilus* = 25.00; *M. marsaeus* = 26.50; *M. satevis = 33.28*). Indeed, *M. mothone* was the only species which readily accepted the *T. speciosa* as a host plant even when no other host plants were provided (H = 47.422, df = 3, *p* < 0.001; Fig. 2). Although *M. satevis* showed a trend of higher acceptability of *T. speciosa* as a host plant, this was not significantly different from the two other species (H = 2.637, df = 2, *p* = 0.104).

**Fig. 3:**
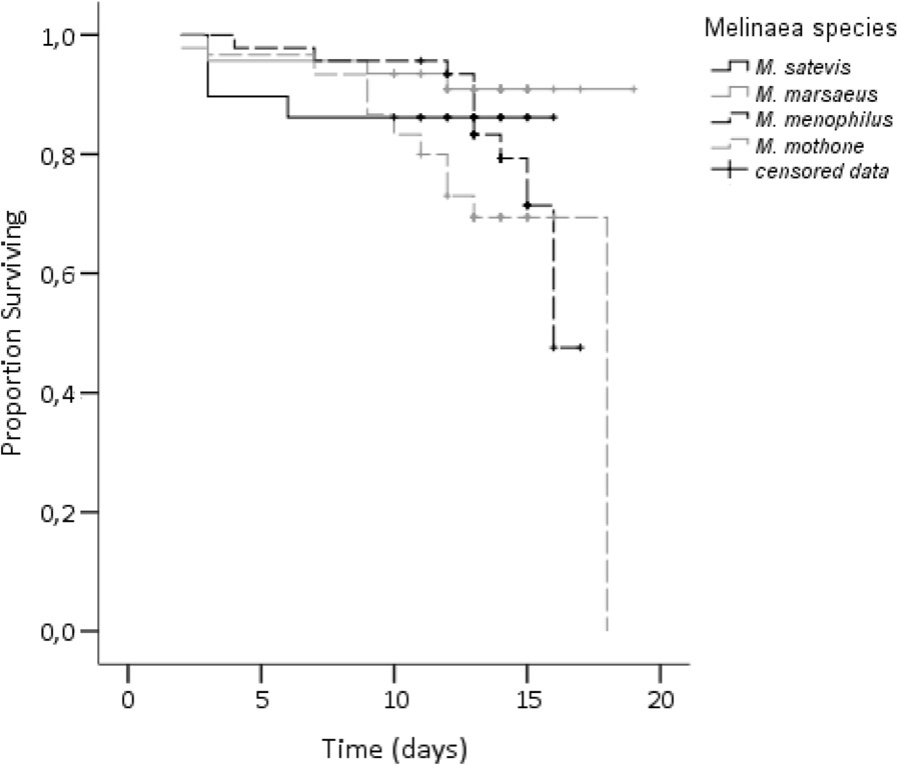
Survival curves for larvae of four *Melinaea* species (*M. menophilus*, *M. mothone*, *M. marsaeus*, and *M. satevis*) reared on *Juanulloa parasitica* (*N* = 32 larvae/species). Censored data indicates survival and butterfly emergence

**Fig. 4:**
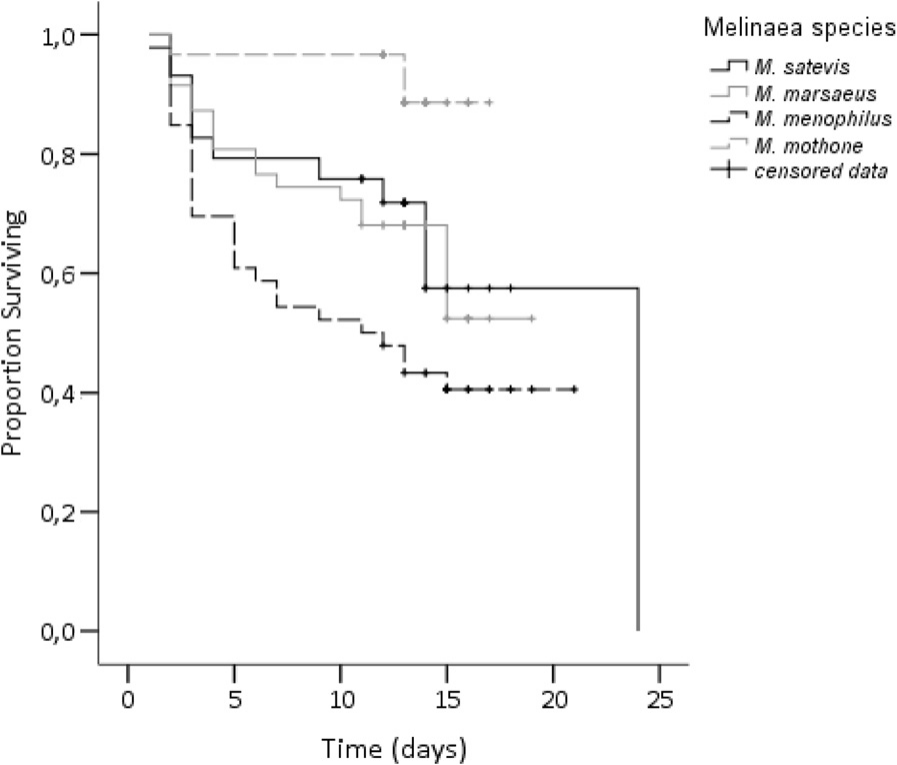
Survival curves for larvae of four *Melinaea* species (*M. menophilus*, *M. mothone*, *M. marsaeus*, and *M. satevis*) reared on *Trianaea speciosa* (*N* = 32 larvae/species). Censored data indicates survival and butterfly emergence

Survival was highest for those larvae reared on *J. parasitica* (average survival rate across species was of 82.0 % on *J. parasitica vs.* 60.9 % on *T. speciosa*). The relative risk of mortality was not significantly different for the different *Melinaea* species (Wald = 5.406; df = 3; *p* = 0.144; Table 1 and Fig. 3), but it was significantly affected by host plant (Wald = 6.086; df = 3; *p* = 0.014; Table 1 and Fig. 4), and there was a significant interaction between the two factors (Wald = 12.662; df = 3; *p* = 0.005; Table 1). This was the result of the higher survival rate of larvae of *M. mothone* on *T. speciosa* (average survival rate of 90.6 %; Wald = 5.790; df = 1; p = 0.016).

**Table 1:** Results of a multivariate Cox proportional hazards models for larval mortality of four *Melinaea* species (*M. menophilus*, *M. mothone*, *M. marsaeus*, and *M. satevis*) reared on either *Juanulloa parasitica* or *Trianaea speciosa* (*N* = 32 larvae/*Melinaea* species/host plant)

Factor	Relative risk	*P*
	(95 % confidence interval)	
*Melinaea* species	1.000	0.144
Host plant	2.074 (1.162–3.701)	0.014
Interaction	1.000	0.005

Most death occurred during the early stages of larval development and it occurred more frequently in larvae reared on *T. speciosa*, except for *M. mothone*, where it occurred equally in both groups. Mean age at death was of 9.9 days for all species reared on *J. parasitica*. However, all species other than *M. mothone* reared on *T. speciosa* died at a much younger age (6.7 days *vs.* 9.3 days for larvae of *M. mothone* reared on *T. speciosa*).

**Fig. 5:**
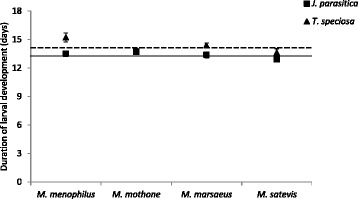
Duration of larval development (Mean ± SE; *N* = 32 individuals/*Melinaea* species/host plant) for four *Melinaea* species (*M. menophilus*, *M. mothone*, *M. marsaeus*, and *M. satevis*) reared on either *Juanulloa parasitica* (*squares*) or *Trianaea speciosa* (triangles). *Full and dash lines* indicate average duration across species on *J. parasitica* and *T. speciosa*, respectively

The average larval development time for all species was of 13.4 days on *J. parasitica* and 14.1 days on *T. speciosa*. Larval development was significantly affected by both host plant (F = 16.396; df = 7, 185; *p* < 0.001) and species (F = 4.030; df = 7, 185; *p* = 0.008), but there was no significant interaction between the two factors (F = 2.331; df = 7, 185; *p* = 0.076; Fig. 5). All species took longer to develop on *T. speciosa* than on *J. parasitica*, except for *M. mothone*, which did equally well on both host plants.

**Fig. 6:**
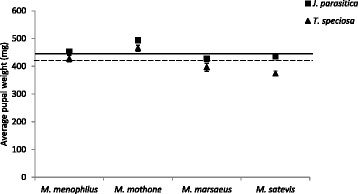
Pupal weight (Mean ± SE; *N* = 32 individuals/*Melinaea* species/host plant) of four *Melinaea* species (*M. menophilus*, *M. mothone*, *M. marsaeus*, and *M. satevis*) reared on either *Juanulloa parasitica* (*squares*) or *Trianaea speciosa* (*triangles*). *Full and dash lines* indicate average weight across species on *J. parasitica* and *T. speciosa*, respectively

Average pupal weight was highest for larvae reared on *J. parasitica* (450.66 mg on *J. parasitica vs.* 420.01 mg on *T. speciosa*). Both host plant (F = 22.558; df = 7, 185; *p* < 0.001) and species (F = 21.129; df = 7, 185; *p* < 0.001) significantly affected mean pupal weight, but there was no interaction between the two factors (F = 1.170; df = 7, 185; *p =* 0.323; Fig. 6). All species weighed less when reared on *T. speciosa.* However, this difference was the least apparent in *M. mothone*.

## Discussion

Determining which traits and corresponding selective pressures initiate divergence is key to understanding the causes of ecological speciation, and diversification in phytophagous insects has often been thought to be driven by shifting and adapting to new host plants [1]. Although the genus *Melinaea* is characterized by a recent and rapid diversification across much of the Neotropics [8–11], we found that most species in this study co-occur with and utilize the same host plant, *J. parasitica*, suggesting that diversification and speciation in this genus has mostly occurred without changes in host plants. We did, however, find large differences in performance on the novel host plant *T. speciosa* between the co-occurring *M. mothone* and the other species, consistent with local adaptation to host plant species.

But the fact that *M. mothone* has retained the ability to use and do equally well on both *J. parasitica* and *T. speciosa*, despite the fact that the former does not co-occur, and in addition to the absence of oviposition preference for the co-occurring host species, suggests that the use of a novel host plant is more likely due to niche expansion as a consequence of plasticity for potential hosts. We also found a positive relationship between oviposition preference and larval performance for the other *Melinaea* species, but the reduction in larval performance on the novel host plant *Trianaea* was not as pronounced as the decline in oviposition. Evidence for asymmetry between oviposition preference and larval performance on novel hosts has been documented for other herbivores, as a result of, for example, plant chemistry, competition or enemy-free space, host abundance, larval conditioning during development [19–21]. It has been postulated that oviposition on novel hosts that are suitable for larval development, be it a result of oviposition mistakes or the result of a labile oviposition strategy, might enable host range expansions [21–23]. Shifts by herbivorous insects are sometimes restricted to related plant species, but also occur on unrelated plants, and can be mediated by chemical similarities of the new host, or can be explained by patterns of parallel cladogenesis, and/or increased ecological opportunities (i.e., hosts that are geographically available) (see [24] and references therein). Our results suggest that some potential to oviposit, feed, and survive on the related but novel host plant *Trianaea* is already present in the genus *Melinaea*, which is consistent with the use of multiple hosts by this group of butterflies throughout their geographic range (see [11]).

Furthermore, a key prediction of ecological speciation is that traits that prevent gene flow from eroding adaptation are likely those that evolved early, either directly as the result of adaptive divergence, or as the result of selection preventing the formation of maladaptive hybrids [25–27]. In the genus *Melinaea*, McClure & Elias [11] observed strong assortative mating among taxa, possibly as a result of homogamy for colour pattern, even in very recently diverged lineages such as the *M. marsaeus* subspecies, and even in the absence of host plant differentiation. In this genus, it is likely that plasticity in host use has enabled for a large geographical distribution, which in turn likely exposed populations to a different suite of potential Müllerian mimics. For example, *Melinaea mothone* has distinct elevational preferences and is associated with typically Andean mimicry complexes, including another *Melinaea* species which shares the same colour pattern, *M. isocomma*. Because predation pressure differs for different mimetic communities [28], spatially segregated populations of *Melinaea* butterflies are likely selected to harbour different colour patterns that coincide with those patterns that are most common within their given mimetic environment [29, 30].

Through the maintenance of a spatial mosaic of mimetic colour patterns, predation on Müllerian mimics constrains geographical distribution and allows for different species or subspecies, even those with similar ecological niches, to exist in different regions [31]. Migrants between populations suffer reduced survival because they are non-mimetic outside their habitat and suffer higher levels of predation attacks, which can directly reduce gene flow between populations by lowering the rate of heterospecific encounters [32]. Switches in mimicry can also lead to pleiotropic changes in mate choice, as assortative mating often coevolves with colour pattern, and reinforcement against maladaptive non-mimetic hybrids [33, 34].

Over time, accumulated differences of other ecological aspects, including but not limited to host plant use, will ultimately accumulate, leading to reproductive isolation and speciation. Nevertheless, the expansion of the potential host plant repertoire, rather than host shifts per se, may have been an important driver of diversification, because more potential host plants means a larger area of distribution and, as such, a larger number of potential niches [3, 4, 35].

## Conclusion

Although access to new host plants can provide opportunities for ecological speciation, we did not find that coexisting species of *Melinaea* partition host plant resources as expected if speciation is primarily driven by host plant divergence. Furthermore, although *M. mothone* shows evidence of local adaptation to a novel host plant, *T. speciosa*, which co-occurs, it does not preferentially lay more eggs on or perform better on this host plant than on host plants used by other *Melinaea* species and not present in its distributional range. Rather, it is likely that diversification in this genus is driven by co-occurring Müllerian mimics and the resulting predation pressure, although this is also likely made possible by greater niche diversity as a consequence of plasticity for potential hosts. To understand the causes and consequences of evolution in ecological traits, more studies are needed of groups in which diversification is recent or ongoing, and for which multiple ecological traits are well-described.
